# Loss of Adam10 Disrupts Ion Transport in Immortalized Kidney Collecting Duct Cells

**DOI:** 10.1093/function/zqab024

**Published:** 2021-05-10

**Authors:** Adrienne Assmus, Linda Mullins, Mairi Ward, Ross Dobie, Robert Hunter, Neil C Henderson, John J Mullins

**Affiliations:** University of Edinburgh/British Heart Foundation Centre for Cardiovascular Science, The University of Edinburgh, Edinburgh EH16 4TJ, UK; University of Edinburgh/British Heart Foundation Centre for Cardiovascular Science, The University of Edinburgh, Edinburgh EH16 4TJ, UK; University of Edinburgh/British Heart Foundation Centre for Cardiovascular Science, The University of Edinburgh, Edinburgh EH16 4TJ, UK; University of Edinburgh/British Heart Foundation Centre for Cardiovascular Science, The University of Edinburgh, Edinburgh EH16 4TJ, UK; University of Edinburgh/British Heart Foundation Centre for Cardiovascular Science, The University of Edinburgh, Edinburgh EH16 4TJ, UK; University of Edinburgh/British Heart Foundation Centre for Cardiovascular Science, The University of Edinburgh, Edinburgh EH16 4TJ, UK; University of Edinburgh/British Heart Foundation Centre for Cardiovascular Science, The University of Edinburgh, Edinburgh EH16 4TJ, UK

**Keywords:** cell plasticity, Adam10 knockout, mCCD_cl1_ cell line, kidney cortical collecting duct, mouse SAME model, CRISPR-Cas9, polyploidy

## Abstract

The kidney cortical collecting duct (CCD) comprises principal cells (PCs), intercalated cells (IC), and the recently discovered intermediate cell type. Kidney pathology in a mouse model of the syndrome of apparent aldosterone excess revealed plasticity of the CCD, with altered PC:intermediate cell:IC ratio. The self-immortalized mouse CCD cell line, mCCD_cl1_, shows functional characteristics of PCs, but displays a range of cell types, including intermediate cells, making it ideal to study plasticity. We knocked out *Adam10*, a key component of the Notch pathway, in mCCD_cl1_ cells, using CRISPR-Cas9 technology, and isolated independent clones, which exhibited severely affected sodium transport capacity and loss of aldosterone response. Single-cell RNA sequencing revealed significantly reduced expression of major PC-specific markers, such as *Scnn1g* (γ-ENaC) and *Hsd11b2* (11βHSD2), but no significant changes in transcription of components of the Notch pathway were observed. Immunostaining in the knockout clone confirmed the decrease in expression of γ-ENaC and importantly, showed an altered, diffuse distribution of PC and IC markers, suggesting altered trafficking in the Adam10 knockout clone as an explanation for the loss of polarization.

## Graphical Abstract

**Graphical Abstract:**
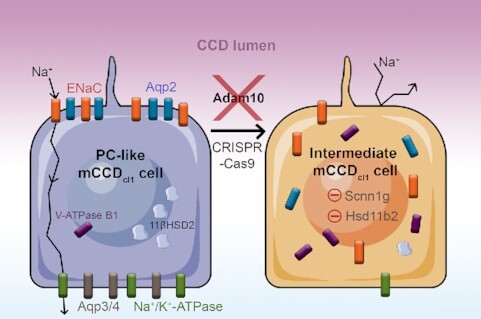

## Introduction

Plasticity has been observed in kidney tubules as an adaptation to mechanical and chemical stimuli. This is observed not only during renal injury but also in healthy adult cells, indicating a capacity for plasticity and regeneration under both pathological and normal physiological conditions. That capacity is particularly evident in the collecting duct, with apparent switching between its two distinct cell types, principal cells (PCs) and intercalated cells (ICs), the latter being subdivided into α- and β-subtypes. Multiple studies have shown that these collecting duct cells are capable of interconversion.^1,2^

Chemical cues may cause collecting duct remodeling: ICs change from β- to α-subtype when subjected to acidic conditions,^3^ treatment with acetazolamide (IC marker CAII inhibitor) leads to an increase in α-ICs at the expense of β-ICs and PCs,^4^ and lithium treatment leads to an increased ratio of ICs to PCs.^5^ These characteristics suggest that plasticity in the collecting duct may be a mechanism for maintaining homeostasis in the kidney, as this segment plays a critical role in the regulation of extracellular volume, pH, and osmolarity. Single-cell sequencing of collecting ducts has identified the presence of an additional “intermediate” cell type between PCs and ICs.^6^

The mechanisms of cell plasticity in the collecting duct have been linked to the Notch pathway, in general associated with the sequential emergence of cell lineage from progenitor cells,^7^ and in particular to nephrogenesis.^8^ The knockout of several factors of the Notch pathway such as *Dot1l*^9^ or *Mib1*^10^ suggests that blocking or downregulating Notch in the collecting ducts generally leads to a decreased number of principal cells and an increased number of intercalated cells, or in the case of Foxi1 knockout to the appearance of the intermediate cell types.^11^ This indicates that the Notch pathway affects cell type determination. For example, the ratio of principal to intercalated cells, typically 70:30 in mice, was adversely influenced by cell-specific knockout of *Adam10* (via deletion of floxed alleles through genetic crosses with Aqp2-cre mice^12^). Adam10 controls the proteolytic processing of Notch and mediates lateral inhibition mechanisms during development.^13^ The apparent disruption of the Notch pathway resulted in a reduced number of principal cells and a corresponding increase in the number of intercalated cells. This was not accompanied by cell death or a significant shift in cell number, strongly suggesting a switch between cell types. Neither the role of cell plasticity nor the underlying mechanism has been completely elucidated in the collecting duct. It is not clear how diseases that disrupt collecting duct function affect cell determination and plasticity, either under normal or pathological conditions such as the syndrome of apparent mineralocorticoid excess (SAME), which is caused by the loss of *Hsd11b2*.^14^ Absence of the enzyme results in increased sodium recovery by the principal cell, mediated through the epithelial sodium channel, ENaC, leading to hypertension, hypokalemia, and pronounced changes to the distal nephron of the kidney.^15,16^

An important in vitro model for collecting duct studies is the mouse cortical collecting duct cell line (mCCD_cl1_), which expresses principal cell factors and proteins enabling physiologically relevant aldosterone-stimulated sodium transport.^17,18^ However, mCCD_cl1_ cells have also been shown to express IC markers, with some intermediate cells expressing both PC and IC markers. The intermediate cells are phenotypically similar to those found in vivo, indicating a capacity for plasticity.^19^ This makes them an ideal system for gene targeting, using CRISPR/Cas9, to interrogate the phenomenon.

We initially studied the composition of collecting ducts from a mouse model of SAME and show important effects of *Hsd11b2* knockout on cell type determination. We then knocked out *Adam10* in mCCD_cl1_ cells, using CRISPR/Cas9 gene targeting. In-depth characterization of the resulting cell lines includes electrophysiological analysis, single-cell RNA sequencing, and immunocytochemical analyses. Here, we report that loss of *Adam10* in mCCD cells led to dramatic effects on sodium trafficking, cell polarization, and PC:intermediate cell:IC ratios, in the absence of major transcriptional changes in the Notch pathway.

## Materials and Methods

### Animal Model and Tissue Preparation

Mice with targeted knockout of the 11β-hydroxysteroid dehydrogenase type 2 (*Hsd11b2)* gene have previously been described^20^ as a model of the SAME. Kidneys were taken from wild-type (WT) and knockout (KO) mice at 4 weeks (*n* = 3) and 17 weeks (*n* = 3), respectively. Kidneys were perfusion fixed, removed, and fixed for further 24 h in 4% paraformaldehyde (PFA), as previously described.^21^Fixed tissue was embedded in paraffin and sectioned at 5 μm thickness, followed by dewaxing, rehydration, and antigen retrieval steps.

### Immunohistochemistry

Primary antibodies and dilutions were as follows: goat anti-Aqp2 (NBP1-70378; Novus Biologicals) at 1:1000 and rabbit anti-V-ATPase B1 (PA535052; Life Technologies) at 1:200. Secondary antibodies used at a 1:500 dilution were donkey anti-goat Alexa Fluor 488 and donkey anti-rabbit Alexa Fluor 568 (A-11055 and A-10042, respectively; Life Technologies).

### Cell Culture

The mCCD_cl1_, mouse cortical collecting duct cell line, was previously established and kindly provided by Bernard Rossier (University of Lausanne, Lausanne, Switzerland). For optimal culture conditions, the cells were cultured at 37°C and 5% CO_2_ in Phenol red-free DMEM/F-12 media (Invitrogen, Life Technologies), with the following supplements: 1 nmol/L triiodothyronine, 5 μg/mL insulin, 50 nmol/L dexamethasone, 60 nmol/L sodium selenite, 5 μg/mL apotransferrin, 10 ng/mL epidermal growth factor (EGF), 2% fetal bovine serum (FBS), and 100 U/mL to 100 μg/mL penicillin–streptomycin (Pen–Strep) as previously described.^17^ During incubation with CRISPR-Cas9 reagents (as per IDT protocol, see later) medium without Pen/Strep was used. Cells were used from passage 28.

### Karyotyping and Fluorescence In Situ Hybridization (FISH) Analysis

Fosmid clones for detection of *Adam10* (WI1-600D17) were selected using UCSC Genome Browser online tool (University of California, Santa Cruz, USA), and obtained from BACPAC Resources (Children's Hospital Research Institute, Oakland, USA). mCCD_cl1_ cells were cultured until confluency, treated for 10 min at 37°C with colcemid (KaryoMAX Colcemid, Gibco), trypsinized and burst (10 min at 37°C) using a hypotonic solution (KCl and sodium citrate). The cell nuclei were then fixed using a 3:1 methanol/acetic acid solution and dropped on microscope slides. FISH analysis was conducted as previously described.^22^

### Immunocytochemistry

On cells fixed in 4% PFA, Adam10 antibody (PA112500, Thermo Fisher Scientific) was used at 1:100 dilution. Rabbit ENaC-γ antibody was provided by the Loffing lab and used at 1:1000 dilution. Goat anti-Aqp2 (NBP1-70378; Novus Biologicals) was used at 1:200 and rabbit anti-V-ATPase B1 (PA535052; Life Technologies) at 1:50 dilution. Identical secondary antibodies as previously described for immunohistochemistry were used.

### CRISPR-Cas9 Targeting

A detailed visual protocol, adapted from the IDT Altr-CRISPR-Cas9 user guide,^23^ can be found in Figure S1. The following reagents were used: CRISPRMAX Cas9 Transfection reagent (CMAX00001, Invitrogen), containing CRISPRMAX and Cas9Plus reagents; Cas9 nuclease (1081058, IDT); Opti-MEM reduced serum medium (11058021, Gibco); tracrRNA (transactivated crRNA) (20 nmol, 1072533, IDT); and nuclease-free duplex buffer (11-01-03-01, IDT). Two different sgRNAs (G1 and G2, 2 nmol, IDT) were designed for Adam10 using the following online resources: UCSC Genome Browser (USA), CRISPR design tool of the Broad Institute (crispr.mit.edu, USA), Benchling (benchling.com, USA), and CRISPR RGEN Tool (rgenome.net/cas-offinder, Hanyang University, Korea) to inform our choice of optimal guides. We recommend choosing two guides in an exon (or neighboring exons) critical to the function of the target protein. If possible, the predicted deletion between the two target sites should not be divisible by 3, so any resulting protein product will be out of frame.

Four guide combinations were tested: negative control (no Cas9 enzyme), G1 (TM containing sgRNA1), G2 (TM containing sgRNA2), and G1+G2 (TM containing a mix of both sgRNA1 and sgRNA2). Technical triplicates were performed for each combination. In brief, guide duplexes were formed by incubating a mix of sgRNA, tracrRNA (transactivated crRNA), and duplex buffer at 95°C for 5 min in a thermal cycler (Veriti 96-well, Applied Biosystems, Foster City, CA). RNP (ribonucleoprotein) complexes were formed by mixing Cas9 enzyme, Cas9 PLUS reagent, Opti-MEM media, and the freshly prepared duplexes, and incubating at room temperature (RT) for 5 min. CRISPRMAX reagent and Opti-MEM media were then added to the RNP complexes, and the solution incubated at RT for 20 min to form the transfection mix. The transfection mix was then added to 200 μL of cell suspension at a concentration of 200 000 cells/mL in growth media lacking Pen/Strep and left to incubate overnight in usual culture conditions described earlier. mCCD_cl1_ cells were trypsinized after 5 days in culture.

Sequence alignment between WT DNA sequence, sgRNAs, and selected primers for polymerase chain reaction (PCR) allowed us to predict sizing of products for each combination (Figure S2).

### Indel Detection and Sequencing

Indels were detected in PCR products using EnGen^®^ Mutation Detection Kit (New England Biolabs). Briefly, heteroduplexes are formed between PCR products with and without indels. The duplexes were then digested using the T7 enzyme and the products analyzed by running the fragments on agarose gel. PCR products were extracted from agarose gels and purified using NucleoSpin^®^ Gel and PCR Clean-up kit (Macherey-Nagel) and sent for sequencing at the MRC PPU DNA Sequencing and Services (University of Dundee, UK). Sequencing results were analyzed with the SnapGene^®^ software (GSL Biotech LLC).

### Cloning

Clonal cell lines were established by serial dilution as previously described^24^ (https://www.corning.com/catalog/cls/documents/protocols/Single_cell_cloning_protocol.pdf). Briefly, confluent mCCD_cl1_ cells in growth medium were trypsinized, suspended in complete culture medium, and serially diluted in a 96-well plate. Following appropriate dilution, the presence of single cells was independently verified and confirmed by observing the growth of the resulting single colonies in the wells over 3 days of culture. The colonies were then trypsinized and transferred to a 6-well plate for culture and screening.

### DNA and RNA Extraction and PCR

Total DNA for each cell line was extracted using DirectPCR^®^ DNA Extraction System (Viagen Biotech). Cells from a well of a 6-well plate were trypsinized, washed twice in phosphate buffered saline (PBS), and pelleted. The pellet was lysed by adding 140 μL of DirectPCR^®^ reagent and 3 μL of proteinase K, mixed thoroughly, and incubated for 4 h at 55°C on a rocking platform, followed by a 45 min incubation at 85°C. The primer sequences for *Adam10* were obtained using PrimerBank, with a WT product size for primer pair 1 of 766 and 458 bp, respectively.

Total RNA was extracted from mCCD_cl1_ cells and KO cell lines using Qiazol (Ambion, Life technologies). cDNA was obtained using 500 ng of RNA with a High-Capacity RNA-to-cDNA kit (Applied Biosystems). The primer sequences for *Adam10* can be found in Table 1. Primers were designed on different exons of the *Adam10* gene and used in different combinations of forward and reverse primer.

**Table 1.** Guide RNAs and Primers for *Adam10* Gene

	Sequence	Additional Information
Adam10 sgRNA 1 (G1)	5′-gaagtgtccctcttcattcgt-3′	Exon 3
Adam10 sgRNA 2 (G2)	5′-gatacctctcatatttacac-3′	Exon 3
Adam10 F1^a^	5′-gccatttatccacatttcctgcag-3′	Exon3
Adam10 R1	5′-cagggaagccggagtgg-3′	Product size: 766 bp
Adam10 F2	5′-gggaagatggtgttgccgac-3′	Exon 1
Adam10 R2	5′-gtgccaccacgagtcttgatg-3′	Exon 4
Adam10 F3	5′-agcgtgccaaacgagcag-3′	Exon 2
Adam10 R3	5′-cctttcaaaaacggagtgatctgcac-3′	Exon 5

a F for forward, R for reverse

The reactions were carried out in a thermal cycler (Veriti 96-well, Applied Biosystems, Foster City, CA), and the amplified PCR products were separated by electrophoresis in a 1.5% agarose gel.

### Electrophysiology

WT mCCD_cl1_ cells and clonal *Adam10* KO mCCD_cl1_ lines were polarized by growing cells on Corning Costar Snapwell Permeable Support inserts (12 mm, 0.4 μm pore size). Cells were seeded at a 1:1 split ratio and grown for 10 days. On day 8, the cells were fed with basal medium containing charcoal-stripped FBS and Pen–Strep supplements only and on day 9 with basal media containing Pen–Strep only. Measurements for transepithelial voltage (*V*_te_) and transepithelial resistance (*R*_te_) were made with a transepithelial volt-ohm-meter and a set of chopstick “STX” electrodes (EVOM2; World Precision Instruments, Sarasota, FL), and the equivalent short-circuit current (*I*_sc_) was calculated using Ohm's law. By convention, a negative *I*_sc_ reflects either electrogenic secretion of cations, electrogenic absorption of anions, or a combination of both. Aldosterone and amiloride (Sigma Life Science) were used at 3 nm and 10 μm, respectively.

### Preparation of Cells for 10X scRNA Sequencing

Cells were prepared as follows: mCCD_cl1_ and each clonal line to be sequenced were seeded on four Corning Transwell filters and cultured for 9 days with complete media. A separate control plate with the same mCCD_cl1_ cells was used for electrophysiological measurements to verify culture conditions through the development of a typical resistive monolayer of mCCD_cl1_ cells. On day 9, cells from 4 filters were trypsinized and resuspended gently, pooled, and the cell suspension diluted in chilled fluorescence-activated cell sorting (FACS) buffer (PBS with 2% fetal calf serum) to obtain approximately 1 million cells in 0.5 mL of buffer. Live–dead cell count was assessed by 4′,6-diamidino-2-phenylindole stain (DAPI-UV excitation 360 nm; emission filter 450/50), and singlets (FSC-A versus SSC-A) were gated to obtain 100 000 live cells using a flow cytometer (BD FACS Aria II SORP, Beckton Dickenson, Basel, CH).

### 10x Chromium Single Cell Library Workflow

Single cells were processed using the Chromium Single Cell 3′ Library and Gel Bead Kit v2 (10X Genomics, PN-120237) and the Chromium Single Cell A Chip Kit (10X Genomics, PN-120236) as per the manufacturer's instructions. In brief, single cells were sorted into PBS + 2% FBS, and washed once. An estimated 7000–10 000 cells were added to each lane of a 10X chip and partitioned into Gel Beads in Emulsion, where cell lysis and barcoded reverse transcription of RNA occurred, followed by amplification, fragmentation, and 5′ adaptor and sample index attachment. Libraries were sequenced with an appropriate platform (HiSeq 4000, Illumina, Inc., San Diego, CA).

Transcriptome libraries with associated UMIs were aligned to the mm10 reference genome (Ensembl 84) using Cell Ranger v2.1.0 Single-Cell Software Suite from 10X Genomics. The resultant datasets were analyzed, both singly and merged, using the Seurat R package v2.4.3^25^ as per the clustering workflow. Briefly, genes expressed in fewer than 3 cells or cells expressing fewer than 200 genes or mitochondrial gene content >30% of the total UMI count were excluded. We normalized using the global scaling “LogNormalize” transformation. Highly variable genes were identified using Seurat's FindVariableGenes function with default parameters. Dimensionality was reduced by principal component analysis (PCA). We performed unsupervised clustering and differential gene expression analyses using Shared Nearest Neighbor (SNN) graph-based clustering, and the first 18 principal components as determined by variability in the PCElbowPlot. The number of clusters was tuned using the resolution parameter. Heat maps, t-distributed stochastic neighbur embedding (t-SNE) visualizations, and violin plots were produced using Seurat functions. Pseudotime between clusters was assessed using the Monocle workflow in R.^26^

Canonical correlation analysis (CCA) was performed on the datasets to look for response to *Adam10* knockout. Correlation strength was assessed by MetageneBicorPlot, and CC1–20 were chosen for analysis. Conserved and differentially expressed genes were identified across the clusters and datasets.

Velocyto 0.17^27^ was run on python to determine the relative proportion of spliced to unspliced transcripts in each library (approximately 20% were unspliced), and the level of unspliced to spliced transcripts for genes of interest. Velocyto R (version 0.6) analysis revealed directionality between cells in t-SNE clusters and also determined the relationship between spliced and unspliced transcripts for genes of interest across clusters.

### Imaging and Image Analysis

Images for FISH analysis were obtained using an epi-fluorescent microscope and associated software (Axioplan 2 microscope and Axiovision software, ZEISS, Oberkochen, DE). The 40×/0.17NA Oil Plan-Neofluor objective was used, and bandpass DAPI, FITC, and GFP filters applied, for detection of DAPI, Alexa Fluor 568, and Alexa Fluor 488 fluorophores, respectively. Immunostaining images were obtained with a QImaging camera (QImaging, Vancouver B.C., Canada) on a fluorescent microscope (Eclipse Ti, Nikon, Tokyo, JP), with DAPI, and TRITC filters applied, for DAPI and Alexa Fluor 568, respectively. The 40× 1.3 NA Plan Fluor oil objective was used. All images were processed and analyzed using ImageJ software (National Institutes of Health, Bethesda, MD). On mice kidney tissue, single channel and composite channel images were analyzed to ensure that all positively stained cells were counted. Secondary verification was applied to a number of images to ensure that the counting process was consistent and unbiased.

Sequencing images were obtained by using the “mapping” and “sequence” functions of the SnapGene^®^ software.

### Statistical Analysis

For mouse tissue data, ratios of cell type were analyzed (PC:IC and IC:intermediate cell) with the GraphPad Prism8 software (GraphPad Software Inc., San Diego, CA). Data are expressed as mean of the log-ratio ± SD. Statistical significance between groups was assessed using a multivariate analysis of variance (MANOVA) test using R software. A *P* < .05 value was determined to be statistically significant. A minimum of 6 images were taken for each sample (prepared section microscope slide). Between 3 and 5 slides were prepared and imaged for each animal (*n* = 3). For electrophysiological measurements, statistical significance was assessed using a Student's paired *t*-test. Data are expressed as means ± SD, and *n* values refer to the number of repeats in an experiment.

## Results

### Effects of SAME on Collecting Duct Composition

**Figure 1.**
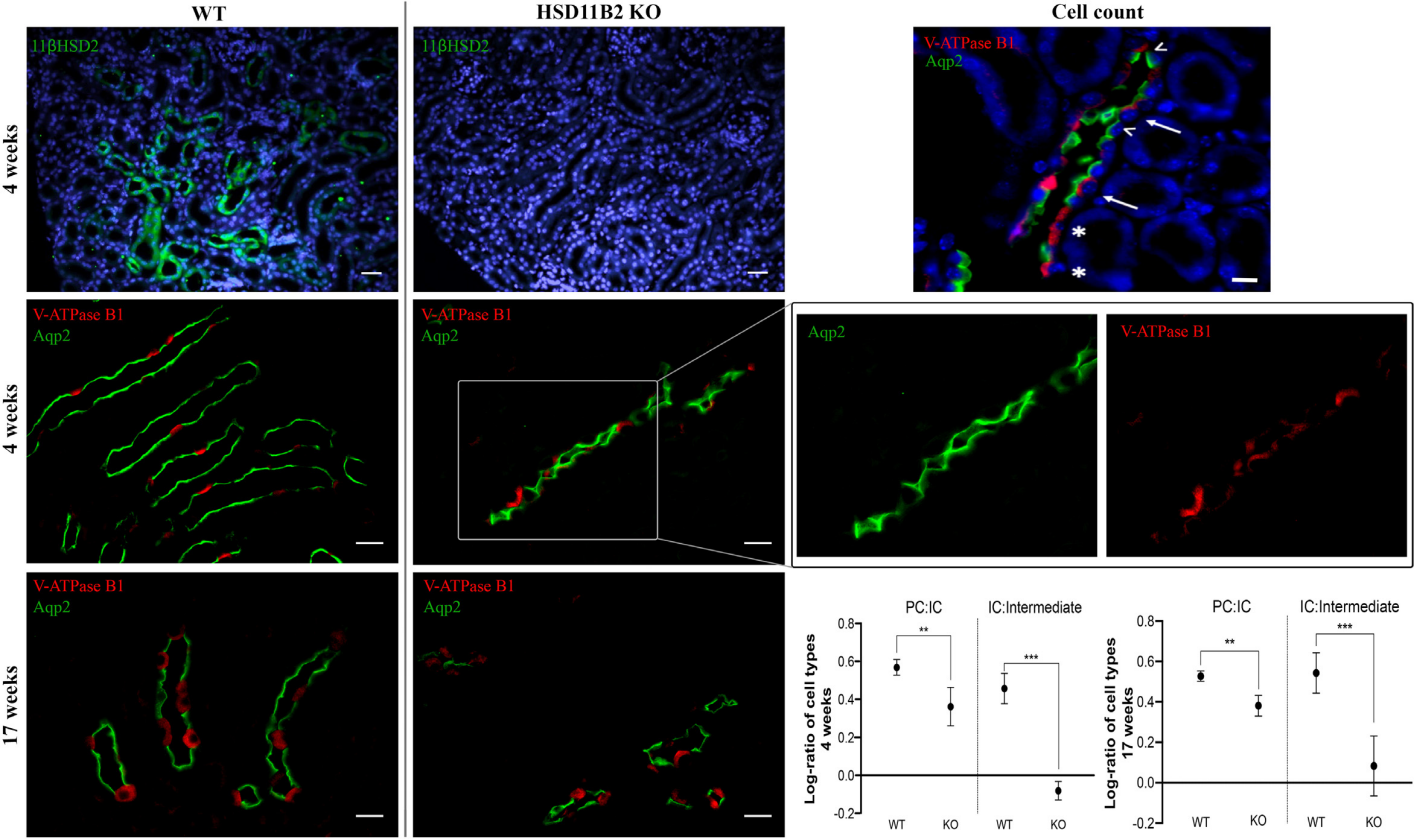
Analysis of CCD Composition in SAME Mice. Top left panel: sample controls for WT and 11βHSD2 KO mice. Epifluorescence images of 5 μm thick kidney sections stained with anti-11βHSD2 antibody (green). DAPI staining of nuclei in blue. Scale bar 30 μm. Middle and bottom left panels: representative images of mouse kidney section stained for Aqp2 (green), and V-ATPase B1 (red) in 4 weeks old and 17 weeks old mice, both WT and KO for the *HSD11B2* gene, with example of separate channels. Scale bars 15 μm. Top right panel: example of staining as used for calculating CD composition in 17 weeks KO mouse; ^ indicates PCs, * indicates ICs, and full arrows indicate double-stained mixed cells. DAPI staining of cell nuclei in blue. Scale bars 10 μm. Bottom right panels: the proportions of PCs, ICs, and intermediate/mixed cells in collecting ducts were counted between groups of WT and *Hsd11b2* KO mice at 4 weeks and 17 weeks (*n* = 3). The log-ratios of PC:IC and IC:intermediate cell are reported as mean ± SD. Significance between groups was assessed using MANOVA test with *P* < .05. ***P < .001, **P < .01.

*Hsd11b2* was detected widely in the 4-week-old WT mouse kidney but was absent in 4-week-old *Hsd11b2* KO mice, confirming the integrity of the knockout (Figure 1). Collecting ducts were identified by staining with antibodies specific for Aqp2 and/or V-ATPase B1 for PCs and ICs, respectively. Cells staining for both Aqp2 and V-ATPase B1 were defined as intermediate cells.

Age-matched male mice at 4 and 17 weeks were used to investigate progression of the phenotype, since sodium handling changes from increased sodium retention to salt wasting at around 80 days.^28^ The log-ratio of PC:IC cells in *Hsd11b2* KO mice differed significantly from those observed in their WT counterparts at 4 weeks (0.36 ± 0.10 versus 0.57 ± 0.04) and 17 weeks (0.38 ± 0.05 versus 0.53 ± 0.03). Similarly, the IC:intermediate cell log-ratio was significantly lower in KO than WT mice at 4 weeks (−0.08 ± 0.05 versus 0.46 ± 0.05) and 17 weeks (0.083 ± 0.15 c.f. 0.54 ± 0.10). Since the relative proportion of IC cells in the CD did not change significantly between WT and KO mice of any age, these results show that loss of *Hsd11b2* results in a shift from PC toward an intermediate cell type.

### Ploidy of mCCD_cl1_ Cells and FISH Analysis

**Figure 2.**
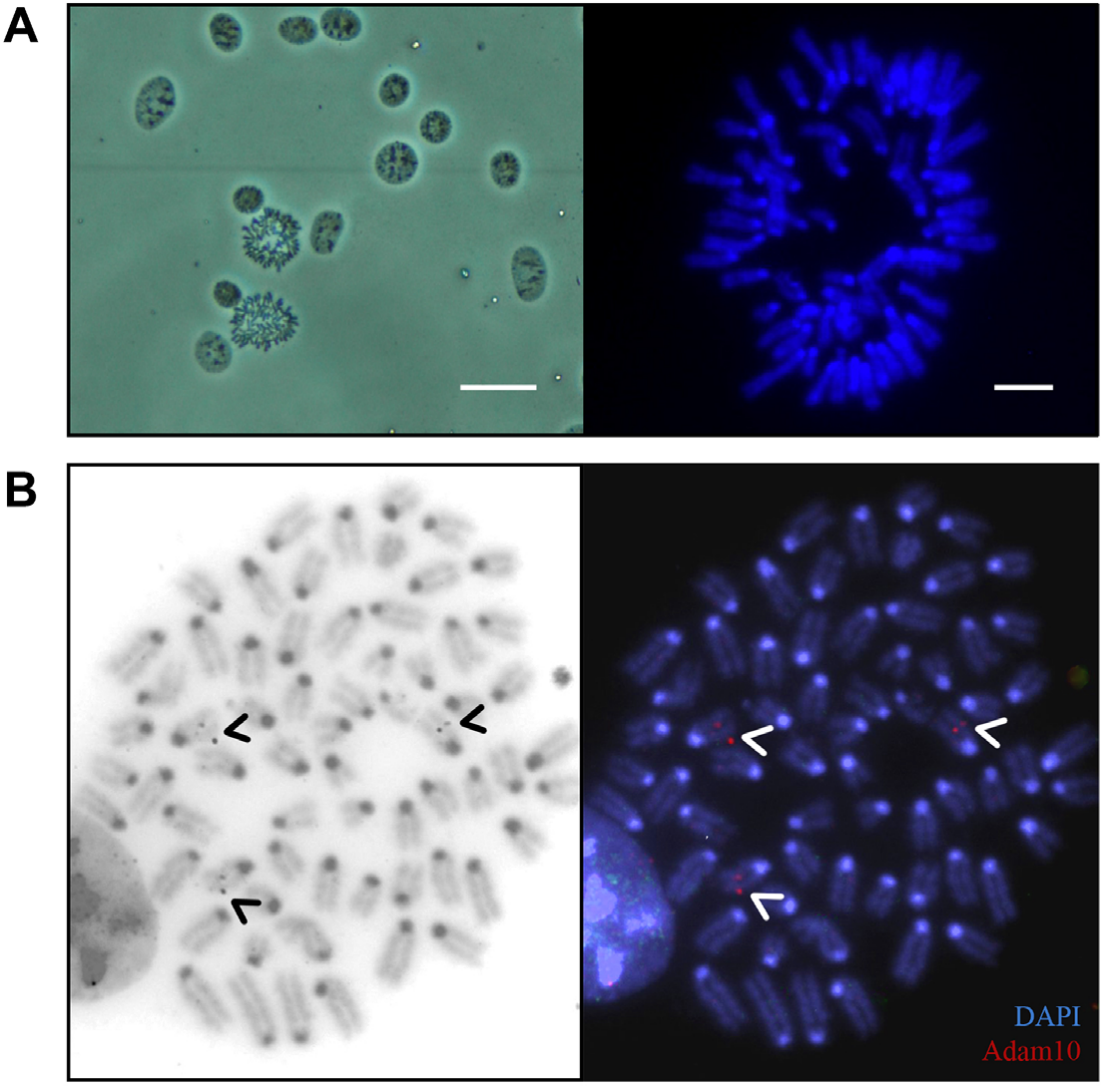
Metaphase Spread and FISH Analysis of Adam10 in mCCD_cl1_ Cells. **(A)** Left panel: example of metaphase spread of mCCD_cl1_ cells. Scale bar 20 μm. Right panel: chromosome spread stained with DAPI for counting. Scale bar 5 μm. **(B)** Representative metaphase chromosome spread tagged with Adam10-specific probes (red channel, left panel). Three chromosomes were identified for the *Adam10* gene (arrows). DNA was stained with DAPI.

While mCCD_cl1_ cells are commonly used for the study of collecting duct physiology, their ploidy has not been reported previously.^18,29–31^ Since ploidy of cells has important implications for efficient gene targeting, we determined the ploidy of mCCD_cl1_ cells, by karyotyping. Chromosome counts from metaphase spreads revealed that the majority of cells contained 64 chromosomes (64 ± 1, *n* = 10) (Figure 2A), suggesting triploidy for the majority of chromosomes, but also some degree of aneuploidy. In order to confirm the number of copies of the genes of interest, FISH analysis was performed. Gene-specific probes were created with fluorescent reporters and incubated on mCCD_cl1_ chromosome spreads. For the *Adam10* gene, situated on chromosome 9, 3 gene-specific probe signals were detected in spreads (Figure 2B). Therefore, complete gene knockout required 3 copies to be targeted.

### Transfection and KO of Adam10 in mCCD_cl1_ Cells

**Figure 3.**
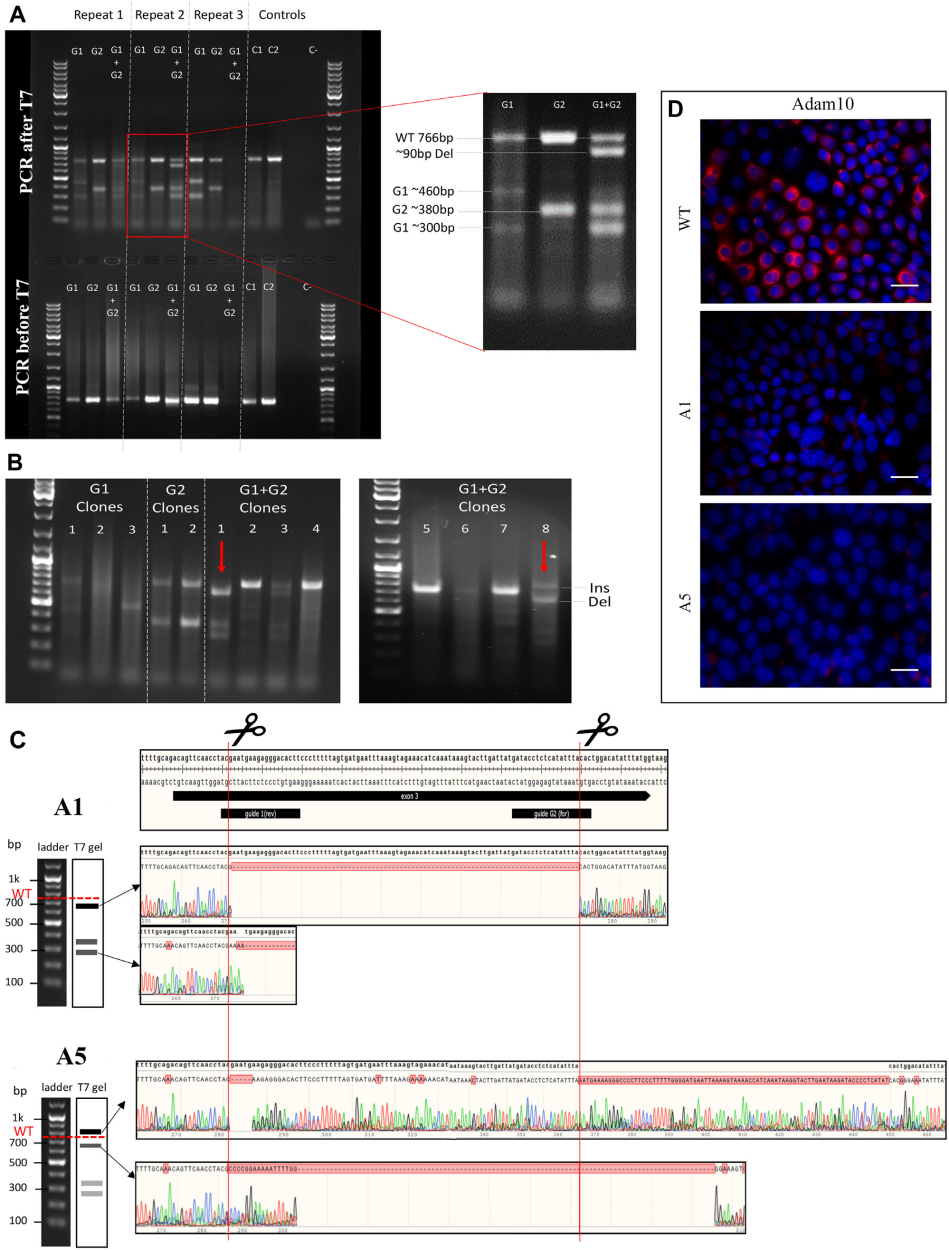
Identification of 2 Clones Resulting From Targeting of the *Adam10* Gene in mCCD_cl1_. **(A)** Transfection results on mCCD_cl1_ cell population for the *Adam10* gene. “Del” for deletion; WT represents the predicted size of the WT product. T7 product of G2 is a doublet. **(B)** T7 screening for Adam10 transfected mCCD_cl1_ cells populations, following single-cell cloning. Red arrows indicate the clones selected after screening, based on the disappearance of a WT-sized band. “Ins” for insertion; “Del” for deletion; WT represents the predicted size of the WT product. The numbers above each gel track are the initial identification numbers of each clone. **(C)** Sequencing of PCR products from both clonal cell lines for Adam10. **(D)** Immunostaining of mCCD_cl1_ cells using anti-Adam10 antibody in wild-type mCCD_cl1_ cells (WT) and KO clonal lines A1 and A5. Composite image with DAPI staining of cell nuclei (blue). All scale bars 30 μm.

CRISPR targeting was achieved using sgRNAs, either singly or in combination. Targeting efficiency was assessed on the whole cell population by PCR (with F1 and R1; see Table 1), and T7 enzyme digestion of the PCR product was used to detect indels. Predicted product sizes for each targeting event for Adam10 were determined (Figure S2). PCR products lacking the expected WT product at 766 bp (Figure 3A) indicate the clone has a high probability of total KO. Two clones were identified from the G1+G2 combination (a 1:4 ratio for G1+G2 clones) and were named A1 and A5 for further analyses. No complete loss of parental PCR product was observed in clones targeted with G1 or G2 alone.

PCR products for each clone, both before and after T7 digestion, were sequenced and revealed a range of editing events (Figure 3C). More specifically, A1 showed a deletion between G1 and G2 loci as well as indels at one, or both target sites, leading to truncated DNA products after T7 digestion. A5 showed a similar deletion event leading to a shorter PCR product compared with WT, as well as an insertion of random DNA sequence at G2 and a short 4 bp deletion at G1, leading to a longer PCR product than WT. Protein expression was assessed in both the parental mCCD_cl1_ cell line and each clone, using immunocytochemistry (Figure 3D). Adam10 could be detected in the WT mCCD_cl1_ cell line. KO cell lines showed a 91.2% ± 4.6% decrease in Adam10 expression for A1, 95.5% ± 3.4% for A5, based on gray value of fluorescence intensity over a set surface (*n* = 8).

### Electrophysiological Characterization of Adam10 KO Cells

**Figure 4.**
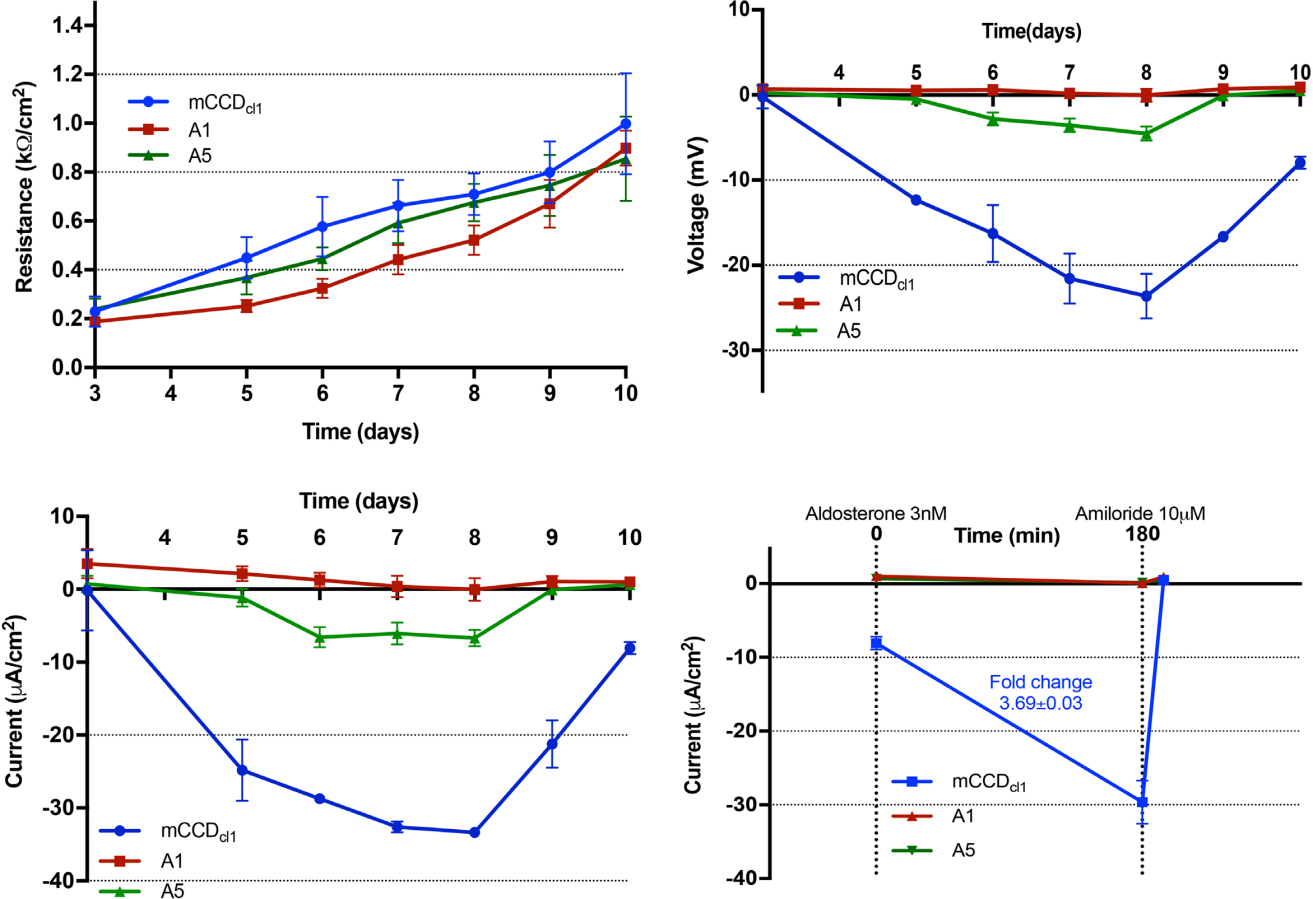
Electrophysiological Analysis of *Adam10* KO Clones. Top left: transepithelial resistance (*R*_te_) measured across monolayers of mCCD_cl1_, A1, and A5 cells grown on Snapwell filters, between days 3 and 10 after seeding. Top right: transepithelial voltage (*V*_te_) measured across monolayers of mCCD_cl1_, A1, and A5 cells. Bottom left: short-circuit current (*I*_sc_) was calculated using Ohm's law. Bottom right: effects on baseline *I*_sc_ of aldosterone (3 nm) and amiloride (10 μm, apical bath) added at *t* = 0 and *t* = 180 min, respectively. Values are shown as means ± SE (*n* = 3).

We next assessed the functional effects of Adam10 knockout. As seen in Figure 4, transepithelial electrophysiological measurements of WT mCCD_cl1_ cells revealed baseline *I*_sc_ measurements of −8.1 ± 0.9 μA/cm^2^ (*n = *3), consistent with previous reports.^17–19^ The application of amiloride (10 μm, 10 min) to the apical bath totally inhibited *I*_sc_, indicating that the basal current can be attributed to the transport of Na^+^ via ENaC. The addition of aldosterone (3 nm, 3 h) increased *I*_sc_ by a factor of 3.7 ± 0.3 fold, to reach values of −29.6 ± 2.9 μA/cm^2^ (see Table 2 for all values). In comparison, identical measurements performed on A1 and A5 show comparable *R*_te_ to WT (values at day 10), but *V*_te_ failed to develop over the course of the experiment, particularly in clone A1. Both KO clones displayed negligible *V*_te_ at days 9 and 10, in media without additives (0.9 ± 0.2 and 0.5 ± 0.0 mV for A1 and A5, respectively). Baseline *I*_sc_ was close to 0 for both KO clones, with a negligible effect of aldosterone and amiloride treatment. In summary, KO of *Adam10* greatly impairs the transport of Na^+^ via ENaC.

**Table 2.** Electrophysiological Measurements for mCCD_cl1_ Cell Line and Clonal KO Lines A1 and A5, and Calculated *I*_sc_ Fold Change After Aldosterone Treatment

	Baseline ± SD	Aldosterone 3 h ± SD	Amiloride 10 min ± SD	*I*_sc_ Fold Change, Aldosterone Treatment ± SD
mCCD_cl1_				
*I*_sc_, μA/cm^2^	−8.1 ± 0.9	−29.6 ± 2.9	0.5 ± 0.3	
*R*_te_, kΩ.cm^2^	1.0 ± 0.1	0.8 ± 0.1	1.5 ± 0.2	3.69 ± 0.03
*V*_te_, mV	−7.9 ± 0.7	−23.3 ± 1.8	0.7 ± 0.3	
A1				
*I*_sc_, μA/cm^2^	1.1 ± 0.3	0.1 ± 0.3	0.9 ± 0.4	
*R*_te_, kΩ.cm^2^	0.9 ± 0.1	1.0 ± 0.1	1.2 ± 0.2	N/A
*V*_te_, mV	0.9 ± 0.3	0.1 ± 0.4	1.0 ± 0.3	
A5				
*I*_sc_, μA/cm^2^	0.7 ± 0.1	0.1 ± 0.4	0.5 ± 0.1	
*R*_te_, kΩ.cm^2^	0.9 ± 0.2	0.8 ± 0.2	1.0 ± 0.2	N/A
*V*_te_, mV	0.5 ± 0.1	0.2 ± 0.3	0.5 ± 0.2	

Abbreviation: N/A, not applicable.

### scRNAseq Analysis of Adam10 KO Clone

To uncover the mechanisms underlying the loss of sodium transport following knockout of *Adam10*, mCCD and A1 clone were grown on Corning Transwells for 9 days, to allow polarization, and were FAC sorted for single-cell RNA sequencing, following the 10X Genomics protocol.

**Figure 5.**
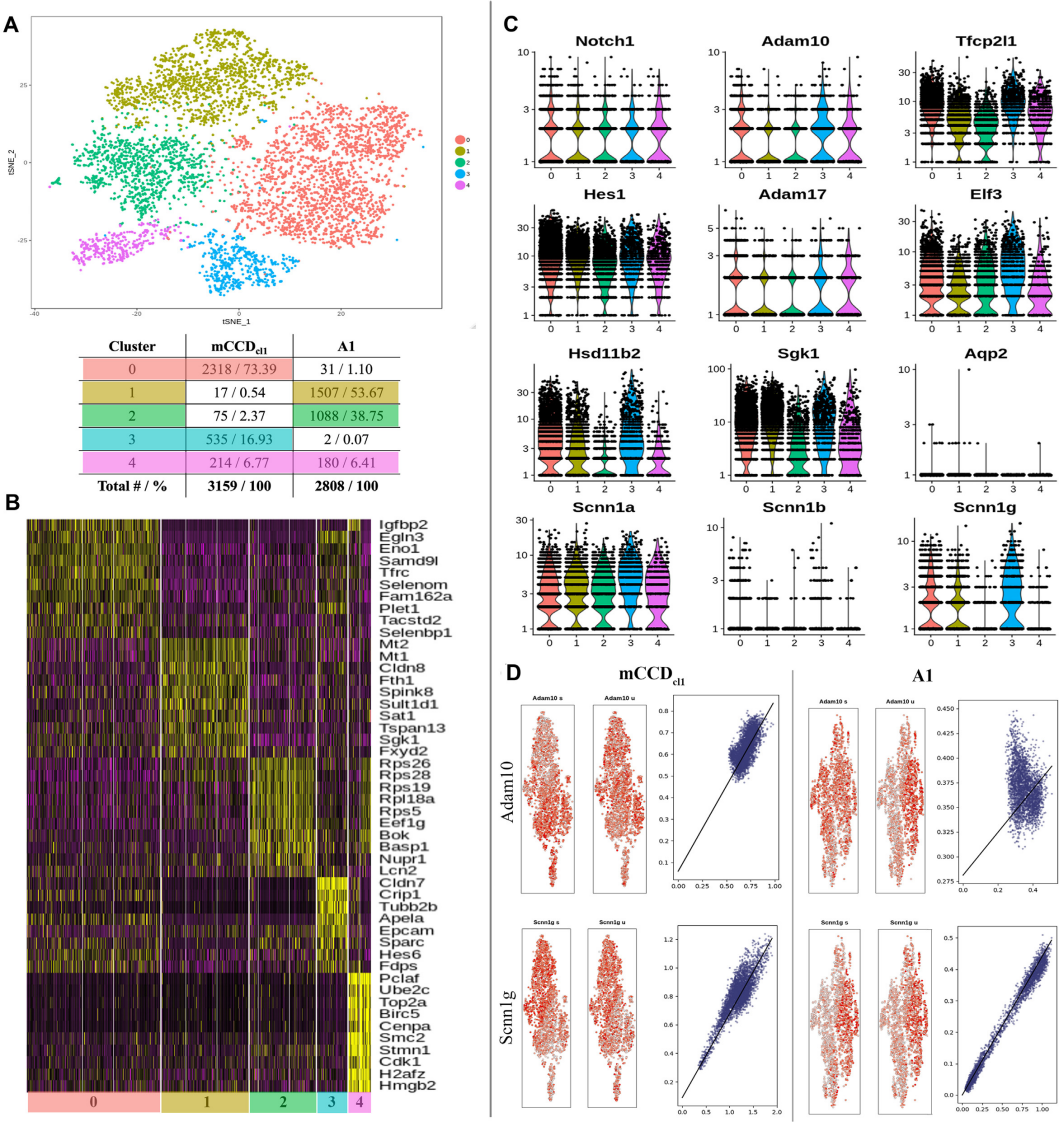
Principal Component Analysis of mCCD_cl1_ and A1 Clone. **(A)** Clusters in different colors are formed by comparing sequencing data from each cell, represented by individual dots. The corresponding table indicates the number of cells (#) and percentage (%) from each cell line in each cluster. **(B)** Heat map of the 10 most informative genes per cluster. **(C)** Violin plots of key markers of Notch pathway and principal cells. **(D)** Velocyto analysis of spliced versus unspliced transcripts of Adam10 and Scnn1g in separated parental and A1 libraries. Left-hand double panels in red show average of 10 cells per circle; right-hand panels in blue plot spliced (s) versus unspliced (u) transcripts.

The transcription profile of A1 was compared with parental mCCD_cl1_ cells by pooling the data while retaining source identity. We first performed linear dimensional reduction by PCA, and then clustered cells of closely related gene expression profile using a t-SNE plot. Figure 5A shows unsupervised clustering of pooled data from the two cell lines (resolution 0.1), resulting in the identification of 5 clusters. The accompanying table identifies the composition of these clusters (mCCD_cl1_, A1). Average gene expression levels per cluster are provided (Dataset S1: PCA Cluster Average). Cluster 4 expresses high levels of cell cycle (cell division) related genes and is a consistent mix containing between 6.0% and 7% of cells of each line. The majority of WT mCCD_cl1_ cells are in cluster 0 (73.4%) and cluster 3 (16.9%), while the majority of A1 cells are in cluster 1 (53.7%) and cluster 2 (38.7%).

Figure 5B shows a heat map of the 10 most differentially expressed genes for each cluster. Notable genes, highly downregulated in the knockout clone, include *Tfrc* (transferrin receptor 1, regulation of intracellular iron levels) and *Crip1* (cysteine-rich protein 1, previously linked to blood pressure regulation^32^). *Parm1*, previously identified as a specific intermediate cell marker in WT mice,^6^ is increased in A1 (data not shown). Genes identified in the heat map allowed us to identify cluster 5 as dividing cells.

Violin plots (Figure 5C) revealed the transcript distribution of key components of the Notch pathway and PC-specific genes across clusters. Despite the introduction of indels into the *Adam10* gene, transcripts are still produced (in common with many knockout strategies), but no active protein is predicted from transcript sequences (Figure 3D). Neither *Notch1* nor *Adam10* (which showed a similar pattern of expression across clusters to *Adam17*) was highly expressed in any of the clusters. There was a significant decrease in transcription of the gamma and beta subunits of ENaC (*Scnn1g* and *Scnn1b*) in knockout cell-rich clusters (see violin plots in Figure 5C), while subunit alpha (*Scnn1a*) did not show significant change.

Velocyto analysis allowed us to investigate the distribution of spliced versus unspliced transcripts in the individual datasets (Figure 5D; note that circles represent average of 10 cells). This clearly demonstrated varying degrees of active transcription and splicing of *Adam10* and *Scnn1g* transcripts across cells of each library but at 50% reduced level in clone A1 compared with mCCD_cl1_ cells.

**Figure 6.**
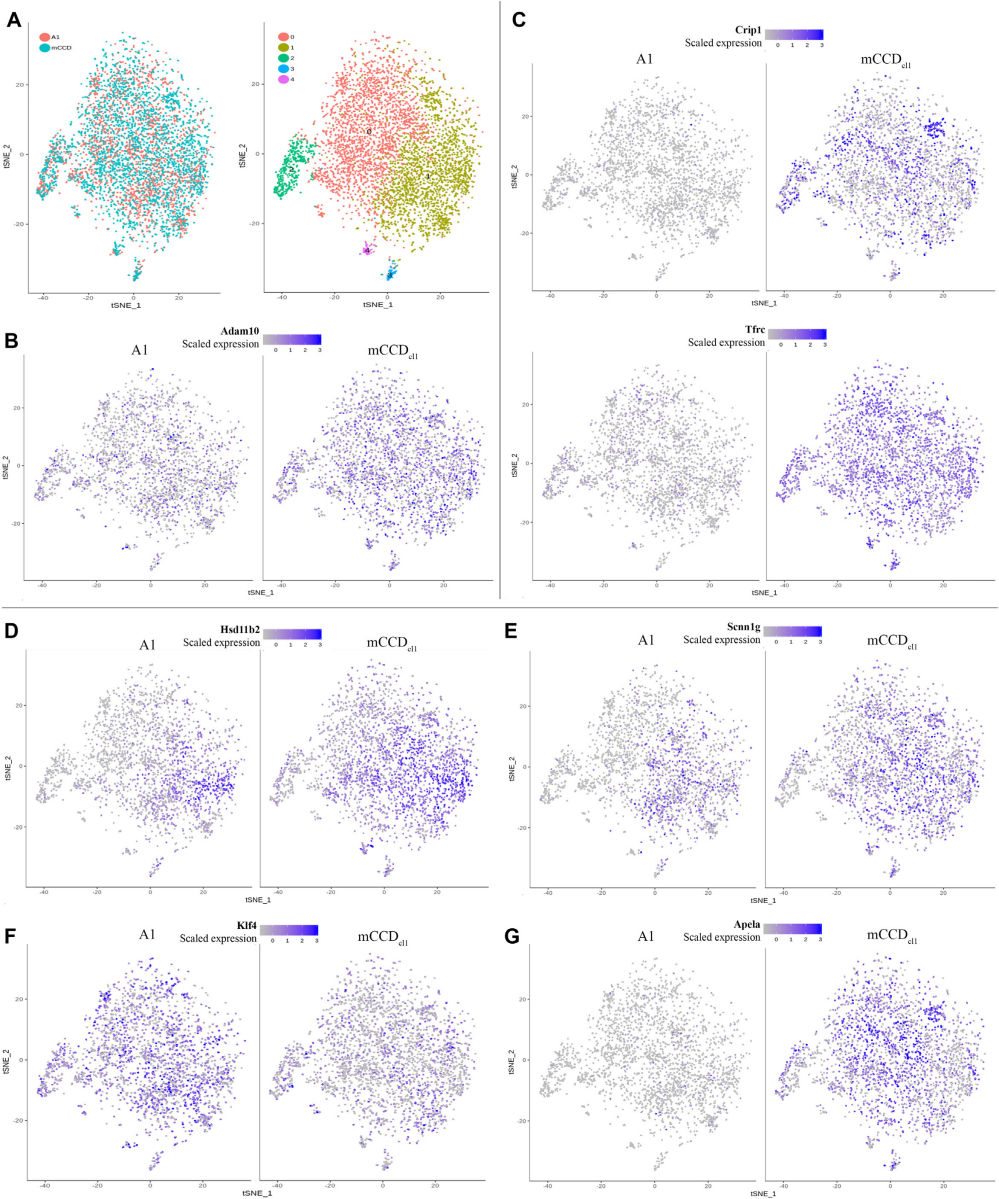
Canonical Correlation Analysis (CCA) of mCCD_cl1_ and A1. **(A)**Visualization of CCA combined libraries projected into maximally correlated subspace before and after cluster analysis (resolution 0.3). Typical FeaturePlots of genes, split according to source library, including **(B)** Adam10 and **(C)** genes *Crip1* and *Tfrc*, downregulated in A1; **(D)** PC-specific genes *Hsd11b2* and **(E)***Scnn1*; **(F)** transcription factor Klf4, upregulated in A1 compared with mCCD, and **(G)** ligand *Apela*, which was downregulated in A1. Positive expression, purple; no expression, gray.

To interrogate the response of clone A1 to *Adam10* knockout, we performed CCA as a means of dimensional reduction. This identifies common sources of variation (such as cell types) in the pooled datasets, and then looks for variation within resolved clusters. The dataset for A1 was mapped closely onto the mCCD dataset using CCA, and five clusters were identified (Figure 6A; resolution 0.3). Feature plots revealed variations in transcript expression between the two libraries, across the clusters. *Adam10* transcription was observed in a smaller percentage of A1-derived cells across the clusters (Figure 6B). Transcription of a number of genes was dramatically reduced in A1, including *Crip1* and *Tfrc*, (Figure 6C), while *Hsd11b2* and *Scnn1g* transcription appeared to be restricted to cluster 1 in A1 (Figure 6D and E) as was *Sgk1*. Of note, a number of transcription factors, including *Klf4* (Figure 6F), showed increased transcription in the knockout clone, while the ligand *Apela*, which is known to regulate fluid balance,^33^ was downregulated (Figure 6G). (The full lists of upregulated and downregulated transcripts in clusters 0 to 4 are given in Dataset S2: CCA A1 Response.)

### Expression and Localization of Key Markers

Considering the reported effect of Adam10 in vivo on the composition of the CCD, further immunostaining and analysis were conducted in clone A1 compared with parental mCCD_cl1_ on markers specific to PCs (γ-ENaC and Aqp2) and ICs (V-ATPase B1).

**Figure 7.**
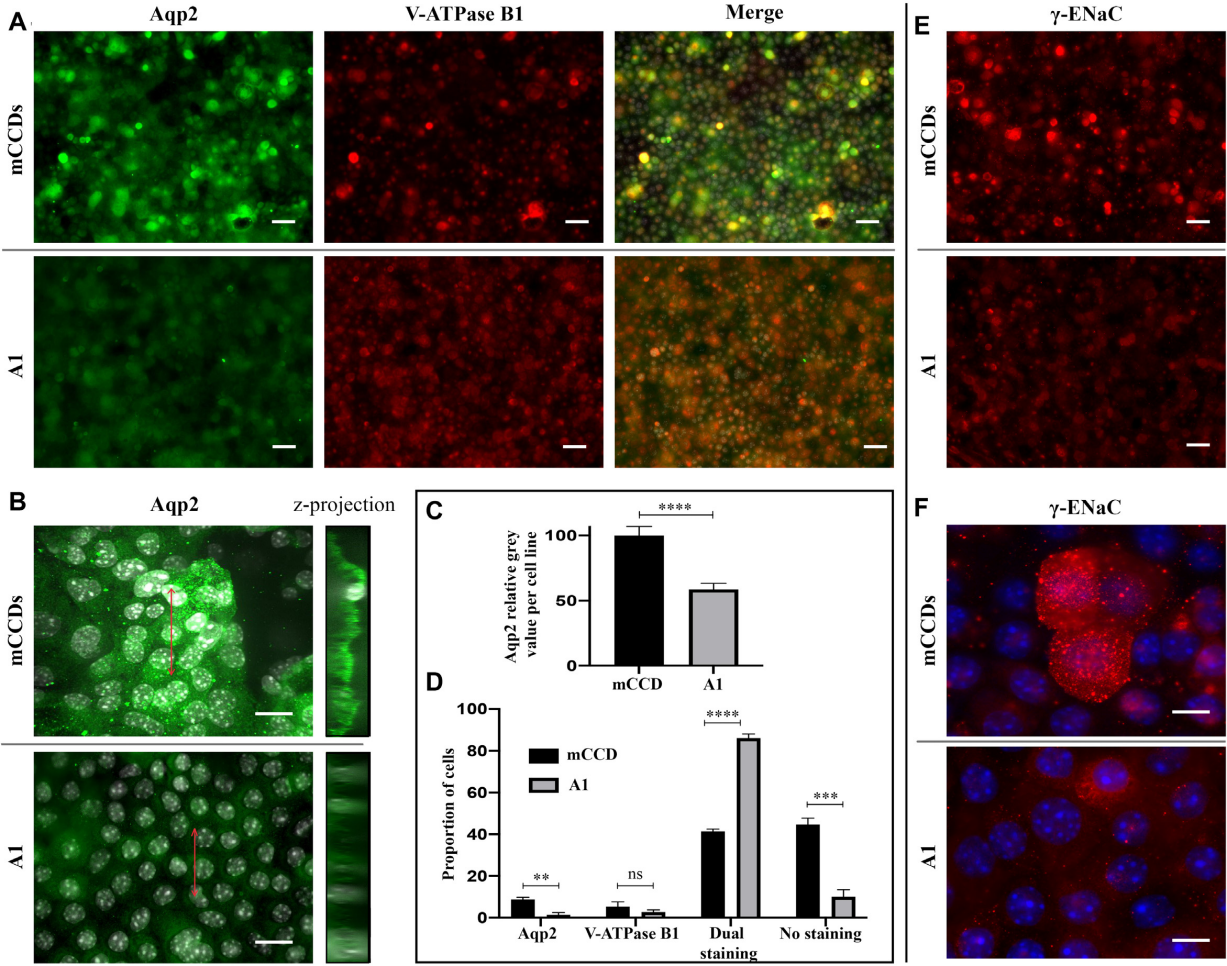
Characterization of A1 by Immunohistochemistry. **(A)** Immunostaining of mCCD_cl1_ cells (mCCDs) and A1 cells (A1) with anti-Aqp2 and V-ATPase B1 antibodies. In the composite images, DAPI staining of cell nuclei is in gray. Scale bars 50 μm.** (B)** Confocal imaging of Aqp2 immunostaining in mCCD_cl1_ and A1 cells. On the right, *z*-projection of the images *z*-stack, localized on the left image by the red arrows. DAPI staining of cell nuclei is in gray. Scale bars 20 μm. **(C)** Gray value per cell line for Aqp2 staining. ^****^P < .0001.**(D)** Quantification of the proportion of cells (%) staining for Aqp2 only, V-ATPase B1 only, both, and neither in mCCD_cl1_ and A1 cells. **P < .01; ***P < .001; ^****^P < .0001. **(E)** Representative images of mCCD_cl1_ and A1 cells stained with anti-γ-ENaC antibody. Scale bars 20 μm. **(F)** Representative images of mCCD_cl1_ and A1 cells stained with anti-γ-ENaC antibody (red), and DAPI in blue. Scale bars 10 μm.

Immunostaining of markers specific to PCs and ICs, Aqp2 and V-ATPase B1, respectively, shows altered expression levels and localization of proteins. Parental mCCD_cl1_ cells showed varied expression levels in individual cells, and a staining distribution in line with previous report^19^: ∼44% of cells with no staining; ∼41% with dual staining of Aqp2 and V-ATPase B1; and a small proportion of cells staining for only Aqp2 (8.7%) or V-ATPase B1 (5.3%). In contrast, A1 showed an overall decrease in Aqp2 expression (−41.4% ± 3.7% in A1) but also a more uniform expression of both Aqp2 and V-ATPase B1 across the whole cell population (Figure 7A, C, and D). Dual staining cells represent 86% ± 2% of A1 cells, mainly due to the decrease in nonstaining cells compared with mCCD_cl1_. Confocal imaging shows that A1 cells lose polarization, with diffuse staining of Aqp2 throughout the cytoplasm, compared with the localization of Aqp2 at the apical membrane in mCCD_cl1_ (Figure 7B).

Equally, γ-ENaC is expressed at low levels but more uniformly throughout the A1 cell population compared with mCCD_cl1_ cells (Figure 7E). High magnification imaging shows typical “punctate” staining of ENaC channels on the apical membrane of mCCD_cl1_ cells, absent in A1 (Figure 7F). These results are consistent with the loss of sodium transport function confirming the electrophysiological assays, and with the lower expression mirrored in the sequencing results.

## Discussion

Knockout of *Hsd11b2* in PCs led to a reduction in PCs and increase in intermediate cells. The functionality of intermediate cells displaying both PC and IC markers in SAME mice is unknown. The reduction in PCs may be a homeostatic mechanism to limit the increase in sodium retention, due to loss of *Hsd11b2* function. The intermediate cell phenotype suggests an undifferentiated state, or dedifferentiation after duct development. The phenotype appears similar to the one described in the work of Blomqvist et al.,^11^ where *Foxi1* gene knockout led to a collecting duct composed entirely of intermediate cells. It has to be noted that the *Foxi1* KO led to renal tubular acidosis, which in turn could lead to a shift of some b-IC to a-IC as shown before in acidic conditions^3^ to manage that acidosis. The *Hsd11b2* knockout mice have previously been reported to display a reduced number of collecting ducts, and additionally, hyperplasia of the distal convoluted tubule^21^ suggesting that potential changes in genomic, environmental, or chemical cues at the collecting duct can affect other parts of the kidney tubules. Our study on SAME mice supports the view that dynamic regulation of the PC/IC ratio is complex and extends beyond genes in the Notch pathway. Though a decreased number of PCs was previously observed in *Adam10* KO mice,^12^ leading to polyuria and hydronephrosis and suggesting a role for Aqp2 in mediating the KO phenotype, very different mechanisms affecting cell plasticity may be at play. Equally, plasticity may be a key response of the collecting duct to various stresses.

Polyploidy, a well-known phenomenon in immortalized cell lines,^34^ has not been reported previously for mCCD_cl1_ cells. FISH analysis of mCCD_cl1_ indicated triploidy for the genes of interest. However, the total chromosome count returned an abnormal number (64 instead of the expected 60 for a triploid cell line), suggesting aneuploidy for some of the chromosomes. Our results show the value of preliminary cell line characterization before undertaking genetic modification work. CRISPR-Cas9-targeted deletion between the two guides, G1 and G2, was the most frequent event observed in knockout clones. Ideally, G1 and G2 should be chosen in an exon (or neighboring exons) critical to the function of the protein. Alternatively, the guides should be chosen so that the resultant deletion produces an out-of-frame product. No total knockout was achieved using a single guide, which may be due in part to the number of clones screened in this study. Using G1+G2, 25% of targeted Adam10 clones were complete knockouts. Since mCCD_cl1_ is polyploid, and each allele is knocked out independently, it is critical to carefully analyze the resultant targeted clones in order to verify complete knockout. Our results indicate total knockout is achieved efficiently using 2 guides and should require only a single round of single-cell cloning and screening. We have used the G1+G2 combination strategy for the knockout of another gene of interest (*Tfcp2l1*, unpublished), where approximately 14% of the clones were completely targeted. This is the strategy that we recommend for total knockout in polyploid cell lines.

The transcription profile of components of the Notch pathway was remarkably consistent between mCCD and clone A1, except for *Tfcp2l1*, which was downregulated, and *Jag1*, which was upregulated in A1. It is possible that knockout of *Adam10* was complemented by *Adam17*, which is expressed at equivalent levels in each cell line and across clusters. Adam17 is known to overlap with Adam10 in its alpha-secretase activity.^35^ RNA sequencing may indicate similar levels of expression of key markers; however, effects on protein trafficking and localization will lead to drastically different phenotypes.

Despite including a significant portion of cells with an intermediate phenotype^19^ (expressing both PC and IC markers), mCCD_cl1_ cells exhibit the expected functions of PCs such as amiloride-sensitive sodium transport. It is not clear whether intermediate cells in vitro (or in vivo) are capable of some physiological function. In this study, the knockout of *Adam10* had dramatic consequences on cell functionality, in particular the capacity to transport sodium. No difference in either *Dot1l* or *Mib1* transcription was observed, suggesting that neither epigenetic effects on ENaC subunit transcription nor protein ubiquitination are likely to explain this observation.

Our results suggest loss of *Adam10* increases the intermediate phenotype ratio, and that the loss of function results from a lack of cell polarization. Immunocytochemistry of A1 suggests that lack of polarization may occur in both intermediate and principal cells, given the diffuse antibody staining observed for Aqp2, V-ATPase B1, and γ−ENaC. Clues to explain the loss of polarization in A1 cells may come from the scRNAseq CCA analysis. *Cldn7* is drastically reduced in A1 compared with mCCD cells. Since Cldn7 is involved in tight junctions, this may have a detrimental effect on cell polarity. Gene lists were analyzed by Gene Ontology (http://geneontology.org/), which revealed enrichment in downregulated genes, encoding proteins involved in plasma membrane rafts, caveola, ER membranes, and focal adhesion (Dataset S2), suggesting a significant change in cell trafficking. This may also be relevant to the *Adam10* knockout mouse.^12^

The importance of cell polarization in the maturation process has previously been reported,^36^ and our data are consistent with these findings. While the role of Notch signaling is now well recognized for the modulation of the PC:IC ratio in the collecting duct, our study shows particular effects of disturbing Adam10 on cell polarization and cellular function that suggest a cellular dedifferentiation phenomenon. In reference to earlier reports on cellular plasticity terminology,^37^ this would suggest that CD cells can “transdifferentiate” through a process of dedifferentiation into an intermediate cell type. The intermediate cell type has indeed previously been observed in adult collecting ducts.^6,38^ Recently, Uchimura et al.^39^ demonstrated the roles played by aldosterone and vasopressin on PC and IC differentiation in kidney organoids, suggesting their importance in collecting duct maturation.

In summary, our study shows that PC:intermediate cell:IC cell ratio is altered in SAME, offers new insights into the functional effects of *Adam10* knockout, and highlights the potential of mCCD cells for understanding collecting duct cell biology. Recognition of the potential cell dedifferentiation occurring under pathological conditions could lead to new targets for treatment of kidney diseases affecting the collecting duct.

## Supplementary Material

zqab024_Supplemental_Data

## Data Availability

The data underlying this article are available in the article and in its online supplementary material. The raw scRNA data are deposited in Edinburgh Datashare, at https://doi.org/10.7488/ds/3022, subject to an embargo until October 12, 2021 during which data can be accessed using the “request a copy” function.
