# Supplementary material for: Loss of Adam10 Disrupts Ion Transport in Immortalized Kidney Collecting Duct Cells
Source: Function (Oxf). 2021 May 10;2(4):zqab024. doi: 10.1093/function/zqab024 (PMC8187228; doi:10.1093/function/zqab024)
Supplement: zqab024_Supplemental_Data [file zqab024_supplemental_data.docx]

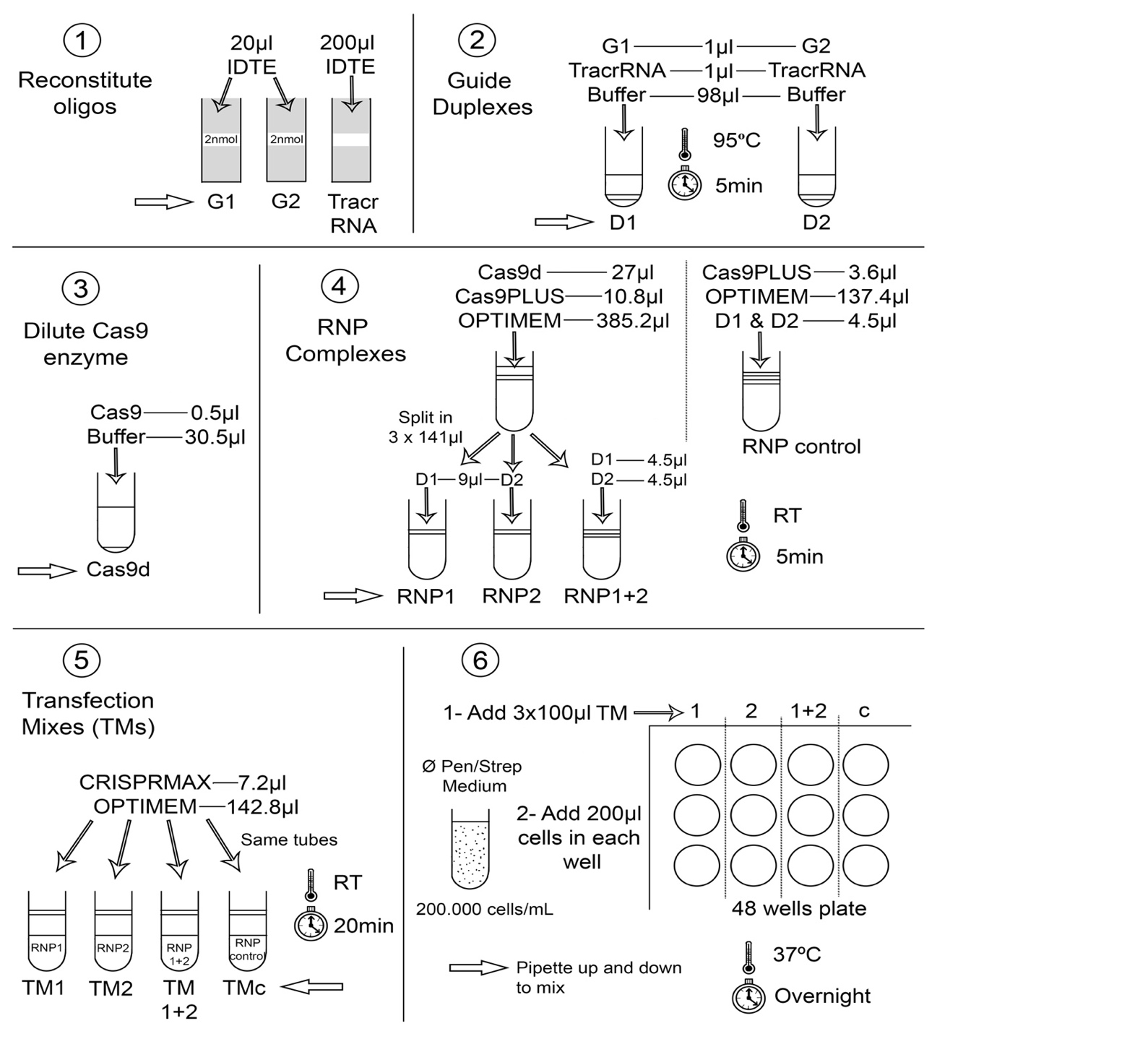


Fig. S1. Detailed visual protocol for CRISPR-Cas9 editing of cells. Adapted from the IDT Altr-CRISPR-Cas9 user guide for use with mCCD_cl1_ cells.





Fig. S2. (a) Predicted product sizes of PCR and T7 enzyme digestion of successful editing events using G1, G2, G1+G2. (b) Visualisation of G1 and G2 localisation on Exon 3 of the Adam10 gene.

Dataset S1 (separate file). PCA cluster average (xlsx file). Average gene expression level per cluster obtained by PCA for mCCDcl1 cell line and A1 clone. First column: gene name. Following columns: values for clusters 0 to 4.

Dataset S2 (separate file). CCA A1 response (xlsx file). Tabs labeled C0 to C4 contain expression data per cluster of genes identified by CCA as the highest variation of transcript expression between mCCD_cl1_ cells and A1 cells. Tabs labeled GO of C0 to C4 are the corresponding Gene Ontology analysis (http://geneontology.org).
